# Implementing the Clean Clinic Approach Improves Water, Sanitation, and Hygiene Quality in Health Facilities in the Western Highlands of Guatemala

**DOI:** 10.9745/GHSP-D-19-00413

**Published:** 2020-06-30

**Authors:** Jason Lopez, Sergio Tumax Sierra, Ana María Rodas Cardona, Stephen Sara

**Affiliations:** a Save the Children, Washington, DC, USA.

## Abstract

**Background:**

Water, sanitation, and hygiene (WASH) services are cornerstones to providing safe health care services and improving patient satisfaction and care seeking. The Clean Clinic Approach (CCA) uses a 10-step process to support health care facilities (HCFs) in making incremental, effective cleanliness and infection prevention and control (IPC) improvements, without relying on external investments. We piloted the CCA in Guatemala and assessed the extent to which it contributed to quality improvements in WASH for IPC.

**Methods:**

After developing an assessment tool tailored to the Guatemalan context, we assessed 11 HCFs in 8 technical areas and scored the facilities on 79 criteria with a total of 100 points. We conducted a baseline assessment (September to October 2018), second assessment (January 2019), and final assessment (February to March 2019).

**Results:**

The 11 HCFs improved their average emergency/general ward scores from 41 points at baseline to 87 points at end line, based on a 100-point scale. For delivery wards, the scores increased from 50 to 91 points and for postnatal wards from 46 to 90 points.

**Conclusions:**

The CCA process and tools facilitated a systematic way for HCFs to identify, prioritize, make, and measure WASH quality of care improvements. Training facility staff was fundamental to improving quality standards, and involving medical and administration staff in joint analysis, coordination, and planning sessions was key to integration and teamwork. Further work is needed to increase involvement of local government and community members and to further adapt the process and tools.

## Abstract (precis)

A water, sanitation, and hygiene (WASH) intervention implemented in a short period in health care facilities with limited resources achieved improvements in health care facility infection prevention readiness.

## Teaser

**Key Findings:**

- Water, sanitation, and hygiene (WASH) services and infection prevention supplies are suboptimal in Guatemalan health care facilities that provide labor and delivery services.
- The Clean Clinic Approach resulted in significant improvements across many WASH and infection prevention readiness indicators despite very little investment.
- Success and sustainment of the Clean Clinic Approach process relies on:
  - Establishing clear, concise, ward-specific standards;
  - Orienting staff to their responsibilities as they apply to the standards provided;
  - Collecting detailed data collection at the ward level; and
  - Sharing survey results with health care facility staff, local governments, and the public.

**Key Implications:**

- To encourage future expansion of WASH in health care facilities, donors and implementing partners should:
  - Collaborate with national ministries of health to review and update national guidelines for infection prevention and control in hospitals,
  - Promote the intervention to hospital directors and regional ministry of health directorates,
  - Partner with health care facility management to update the assessment tools according to level of care and type of service/ward with consideration for basic level of service, and
  - Expand the intervention to include more health care facilities and share the results publicly.

Resumen en español al final del artículo.

## BACKGROUND

A report by the World Health Organization/United Nations Children’s Fund Joint Monitoring Proramme (JMP) stated that worldwide, 26% of health care facilities (HCFs) lack basic water services and 21% lack basic sanitation.^1^ Data from 78 low- and middle-income countries (LMICs) showed that half of 129,557 HCFs lacked access to piped water, 33% did not have an improved toilet, and 39% had no soap for handwashing. In all, 2% of facilities provided complete water, sanitation, and hygiene (WASH) services.^2^

WASH services are vital for providing safe health services, improving patient satisfaction, and improving care seeking. According to a 1995–2008 review and meta-analysis, health care-associated infections developed in more than 15% of patients in limited-resource settings.^3^ Furthermore, the lack of proper infection control in HCFs, including WASH, is a driver for antimicrobial resistance, along with inadequate sanitation and water services in general.^4^ In the world’s least-developed countries, sepsis is responsible for 13.8% of newborn deaths and pneumonia is responsible for 6.1% of newborn deaths.^5^ Neonatal infections in HCFs occur partly from the lack or inadequate delivery of WASH services. Lack of WASH services are negatively associated with patient satisfaction, thus influencing women’s choice for birthing at a facility.^6^

In 2016, 5% of HCFs in Latin America had no water services.^1^ In Guatemala, 33% of HCFs lack 24-hour-a-day water service and only 25% have a corresponding maintenance program. For sanitation, 32% of HCFs lack operational services and 62% had no soap available for handwashing.^7^ Infections cause 26.5% of maternal deaths in Guatemalan hospitals compared to 12.5% of deaths in nonhospital facilities.^8^ Of newborn deaths, 16.8% are caused by sepsis and 5.9% by pneumonia.^5^ Infections also complicate and increase the cost of treating patients. A case control study from a hospital in Guatemala found that the cost of treatment for any given patient with a health care-associated infection was 2.5 times higher than treatment without.^9^

## CLEAN CLINIC APPROACH

**FIGURE 1.**
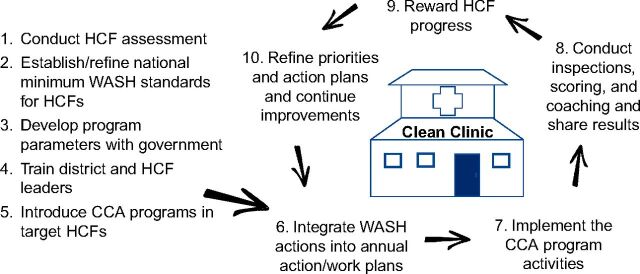
10-Step Clean Clinic Approach for WASH Quality Improvements Abbreviations: CCA, Clean Clinic Approach; HCF, health care facility; WASH, water, sanitation, and hygiene.

The United States Agency for International Development (USAID) Maternal and Child Survival Program (MCSP) developed the Clean Clinic Approach (CCA) to empower HCF staff and health systems to implement simple, low-cost, and effective WASH improvements that are proven to help protect patients and staff from infection. CCA focuses primarily on management, motivation, and accountability as key drivers to maintaining WASH and infection prevention services. This approach is similar to the Plan-Do-Study-Act model for quality improvements that has been successfully used for infection prevention and control (IPC) and has been previously modeled for use in LMICs.^10^^–^^12^ The CCA uses 10 steps to implement incremental WASH and infection prevention improvements to provide quality health care services and prevent health care-associated infections (Figure 1).^13^ Before implementing the approach in Guatemala, MCSP previously piloted the CCA in Haiti.^14^

The CCA acknowledges that HCFs face multiple challenges to improving WASH including include missing, incomplete, or poorly implemented national standards; limited funding; and lack of knowledge or adherence to IPC protocols by health workers.^15^

To mitigate these challenges, the CCA approach encourages collaboration between program implementers and the national ministry of health to develop WASH for IPC evaluation criteria and ratings systems. Then, the CCA implementer works directly with HCFs to improve their rating to meet local standards by developing action plans and making incremental WASH improvements on their own.

The CCA intervention in Guatemala aimed to increase the availability of functional WASH infrastructure and basic infection prevention supplies at HCFs through incremental monitoring and management and behavioral improvements without relying on external investments. Specifically, the intervention aimed to: (1) define a package of quality standards to monitor WASH components used in 11 Ministry of Public Health and Social Assistance (MSPAS) HCFs with delivery care services in the Western Highlands of Guatemala, along with a tool and process for monitoring and supporting progress; (2) serve as a basis for the development of a training curriculum in WASH for hospitals, centers for permanent attention (*centros de atención permanente*, CAPs), and centers for integral attention of maternal and child health (*centros de atención integral materno infantil*, CAIMIs) in Guatemala; and (3) institutionalize the Clean Clinic quality standards, tools, and process within the MSPAS systems.

This case study examines to what extent the CCA intervention improved WASH quality standards for IPC.

## METHODS

In Guatemala, the MSPAS is responsible for upholding the national policies for potable water and sanitation, as well as WASH in HCFs as a whole.^16^ Although national policies on WASH in HCFs existed, tools for monitoring the WASH status of facilities had yet to be developed as of the start of the intervention. CCA implementation began in March 2018 in 11 MSPAS HCFs with delivery care services in the Western Highlands of Guatemala that were selected by MCSP and the MSPAS from MCSP-supported facilities (Table 1 and Table 2).

**TABLE 1.** MCSP Facilities Implementing Clean Clinic Approach, Western Highlands, Guatemala, N=11

**Level of Care**	**Type of Facility**	**No.**	**Description**
Secondary	Centers for integral attention of maternal and child health	2	Provide “normal and “uncomplicated” births Open 24 hours/day Capacity for minor surgeries including cesarean deliveries and postabortion care
	Centers for permanent attention	5	Provide “normal and “uncomplicated” births Open 24 hours/day
Tertiary	District hospitals	3	Open 24 hours/day Capacity for major surgeries
	Regional hospitals	1	Open 24 hours/day Capacity for major surgeries and specialties

Abbreviations: MCSP, Maternal and Child Survival Program.

**TABLE 2.** Clean Clinic Approach Implementation Timeline in Guatemala

	**Date**	**Activities**
2018	January	Initial evaluation of health care facilities
	February	Presentation of initial evaluation results to MSPAS and decision to move forward with CCA
	March	Clean Approach implementation begins
	April	Define quality standards, criteria, and weighting thereof
	May	
	June	Familiarize staff with tool in 11 health care facilities
	July	
	August	
	September	Baseline assessment (first measurement)
	October	Identify gaps and define plans for continuous quality improvement
	November	Supervision of improvement plans, coaching, and mentoring
	December	
2019	January	Supervision of improvement plans and second measurement
	February	Supervision of improvement plans and third measurement
	March	Certification of establishments according to established categories: silver, gold, and diamond
	April	Closures and delivery of recognition to establishments and staff

### Initial Evaluation

The MSPAS and MCSP developed a monitoring strategy based on the WHO standards for improving quality of maternal and newborn care in health facilities while integrating the JMP basic service indicators for WASH in HCFs.^17^^,^^18^ MCSP conducted an initial evaluation in January 2018 of the 11 HCFs across 3 wards: emergency/general, labor and delivery, and postnatal and recovery. This evaluation provided a snapshot of WASH and IPC services to guide monitoring and improvement priorities.^18^

MCSP and USAID presented the initial evaluation results to MSPAS leadership (vice ministry for hospitals and CAPs/CAIMIs) and garnered national support for implementing a pilot CCA intervention.

### CCA Tool Development

Using the results of the initial evaluation, the MSPAS Central Team; the Board of the Comprehensive Health Care System; the Department of Regulation of the Health and Environment Programs of the General Board of Health Regulation, Control, and Surveillance; and the General Coordinator of Hospitals, together with 4 MCSP staff members (2 advisors and 2 specialists; 3 doctors and 1 graduate nurse), formed a working group to develop an assessment tool for quality standards and their respective criteria.

**FIGURE 2.**
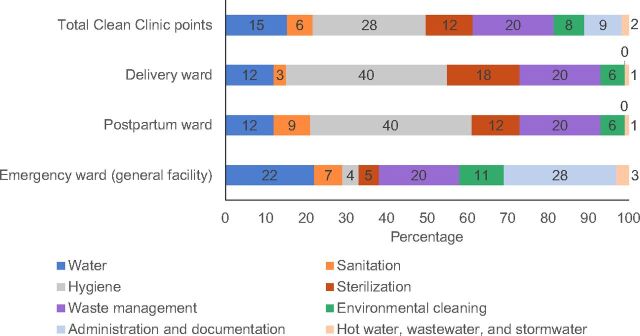
Guatemala Clean Clinic Assessment Scoring Distribution in Total, by Ward and Technical Area^a^ ^a^Total Clean Clinic score is based on the average between wards.

The assessment tool evaluates across 8 technical areas: (1) water; (2) sanitation; (3) hygiene; (4) sterilization; (5) waste management; (6) environmental cleaning; (7) administration and documentation; and (8) hot water, wastewater, and stormwater. The emergency ward criteria also encompassed general facility attributes such as administration or wastewater. The tool consists of 79 criteria, which vary by ward and are weighted according to their impact on IPC, totaling a score of 100 points. Figure 2 provides the scoring distribution for the assessment tool by ward and technical area, and Supplement 1 includes the final tool.

### CCA and Tool Sensitization

MCSP held a workshop with the MSPAS Central Team on using and implementing the newly developed assessment tool that incorporated key national guidelines on controlling and preventing nosocomial infections.^19^ Afterward, MCSP held a workshop with MSPAS regional directorates, municipal government representatives (responsible for the infrastructure of the 5 CAPs and 2 CAIMIs), and directors of the 4 hospitals to outline the CCA and share the preliminary results of 11 HCFs’ initial evaluation.

### Tool Testing

MCSP held a workshop with the MSPAS Central Team and the operational staff of the 11 HCFs (doctors, nurses, sanitation inspectors, rural health technicians, and administrative staff) to provide an overview of the CCA, the assessment tool, and the national guidelines. HCF staff provided feedback on the assessment tools, and some corrections and adaptations were made. Subsequently, MCSP and MSPAS representatives tested the tool in a hospital and a CAP, allowing the team to clarify language and protocols as well as establishing appropriate timing and locations for the application of the tool.

### Health Care Facility Quality Improvements

MCSP and the MSPAS Central Team established “Clean Clinic Teams” at each HCF to jointly perform 3 assessments with MCSP. Using the finalized Guatemala CCA assessment tool, a baseline assessment was conducted across 3 wards in each facility from September 2018 to October 2018 to identify existing gaps in WASH for IPC services. An additional assessment was conducted in January 2019, and a final certification assessment was performed from February 2019 to March 2019 (Table 2).

Clean Clinic Teams at each facility together with MCSP conducted baseline assessments to identify existing gaps in WASH.

#### Quality Improvement Plans

After analyzing the baseline assessment results, the CCTs developed quality improvement plans according to an IPC prioritization matrix to identify and prioritize the problem(s), identify the causes and prioritization of the problem(s), develop/generate possible solutions, and test and implement the proposed changes.

#### Coaching and Mentoring

MCSP conducted WASH for IPC training, coaching, and mentoring on management of water, solid waste, sanitation, and infrastructure for hygiene (Box).

BOX WASH for Infection Prevention and Control Training, Coaching, and Mentoring Topics

- **Water management:** Water supply, storage, and quality
- **Solid waste management:** Segregation and internal and external supply chain
- **Sanitation management:** Cleaning, disinfection, and use of personal protective equipment by the staff
- **Infrastructure management for hygiene:** Toilets, showers, and washbasins of users and health providers and standards of care for infection prevention and control

To facilitate the WASH for IPC trainings, MCSP secured external funding for the relevant materials and supplies (water filters, personal protective equipment, boots, tools, and red hazardous waste bags and labels). These materials and supplies were approximately 4% of the total CCA implementation costs.^20^

#### Assessments and Recognition

Facilities scoring above 70 points were given Clean Clinic certification and were rated as silver (70%–80%), gold (81%–90%), and diamond (91%–100%).

After the certification assessments, MCSP and the MSPAS presented a plaque to each HCF during a public ceremony with the category it reached and gave a diploma to each CCA team member in each HCF.

### Ethical Considerations

The Save the Children USA ethics review committee reviewed the CCA project plan and determined it was exempt from full review.

## RESULTS

Overall, HCFs improved their mean CCA assessment scores from 45.6% at baseline (September 2018 to October 2018), to 73.1% at second assessment (January 2019), to 89.3% at end line assessment (February 2019 to March 2019). Individual ward scores improved with general/emergency wards increasing by 46.2% (from 41.0% to 87.2%), delivery by 40.9% (from 49.7% to 90.6%), and postpartum by 44.2% (from 45.7% to 90.0%). Administration had the most improvement from 0.7% to 7.3%. Cleaning improved the least from 4.5% to 6.5%. Supplement 2 provides detailed results for each facility by assessment number, ward, and sector.

Examining the assessment results through the JMP standards for WASH in HCFs, no facilities met basic service levels for sanitation or waste management at baseline (Table 3).^18^ At end line, all facilities had reached basic levels of service for water and hygiene, and hygiene improved the most.

**TABLE 3.** JMP Classifications for CCA Facilities at Baseline and End Line Assessment, by Ward and Overall Facility (N=11)

**JMP Standards**	**Service Category**	**Overall, No.**		**Emergency, No.**		**Delivery, No.**		**Postpartum, No.**	
		**Baseline**	**End line**	**Baseline**	**End line**	**Baseline**	**End line**	**Baseline**	**End line**
Water	Basic	4	9	3	9	6	10	6	11
	Limited	6	2	6	2	3	1	4	0
	No Service	1	0	2	0	2	0	1	0
Sanitation	Basic	0	5	0	6	1	5	2	8
	Limited	8	5	8	4	5	5	5	2
	No Service	3	1	3	1	5	1	4	1
Hygiene	Basic	2	11	3	11	3	11	2	11
	Limited	7	0	6	0	7	0	8	0
	No Service	2	0	2	0	1	0	1	0
Waste Management	Basic	1	6	1	6	1	6	1	7
	Limited	7	4	7	4	7	4	5	4
	No Service	3	1	3	1	3	1	5	0
Environmental Cleaning	Basic	0	6	0	6	3	6	1	6
	Limited	2	1	2	5	1	5	3	5
	No Service	9	4	9	0	7	0	7	0

Abbreviations: CCA, Clean Clinic Approach; JMP, World Health Organization/United Nations Children’s Fund Joint Monitoring Programme.

Overall, HCFs improved their mean CCA assessment scores by more than 40%.

The following sections describe the results of the clean clinic assessments in total and by the 8 technical areas.

### Total Clean Clinic Assessment Scores

**FIGURE 3.**
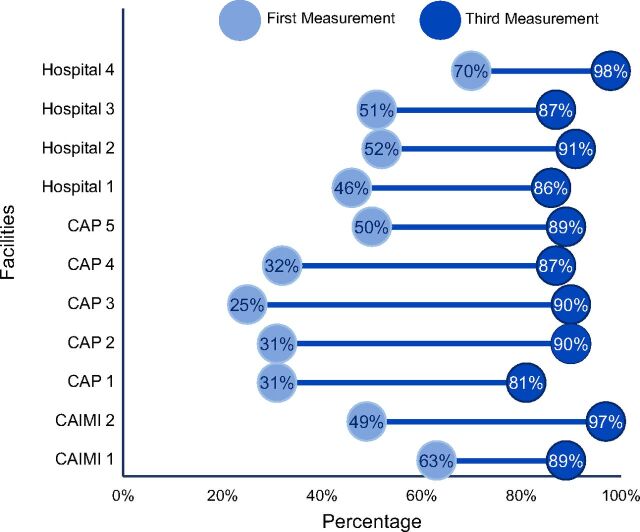
Baseline and End Line Assessment of Overall Compliance Level of Clean Clinic Assessment Criteria Before and After Clean Clinic Approach Intervention, by Facility (N=11)

With the exception of 1 hospital and 1 CAIMI, the CCA facilities had low levels of overall compliance at baseline, with 4 CAPs that had scores below 35% (Figure 3). By the second assessment, compliance levels improved as 5 facilities reached silver status and 2 reached gold status. By end line, all 11 facilities achieved Clean Clinic status: 8 facilities achieved gold certification and 3 earned diamond status.

The closing of gaps in scores between the first and third assessments was most pronounced in the 5 CAPs. On average, the CAPs’ compliance increased 54%, with the highest improving by 65%. The 2 CAIMIs saw an average improvement of 37%, and hospitals improved by 36% (Figure 3).

### Water

**FIGURE 4.**
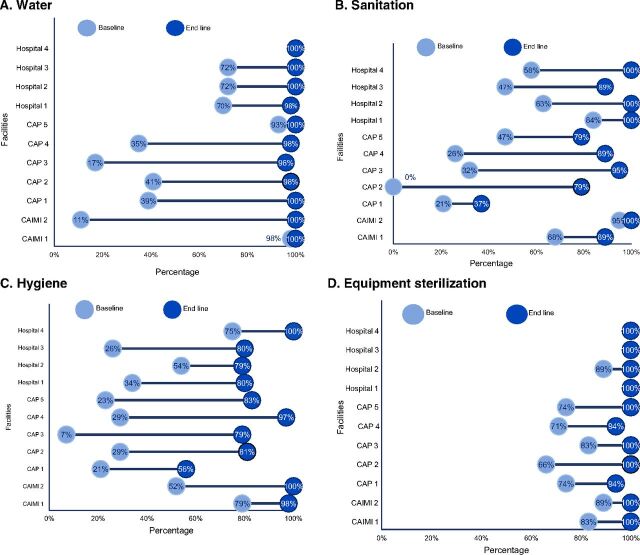
Baseline and End Line Assessment of Facility Compliance Level in Water, Sanitation, Hygiene, and Equipment Sterilization Before and After Clean Clinic Approach Intervention, by Facility (N=11)

The water standard uses 9 criteria and contributes 15 of the 100 Clean Clinic certification points, based on an average across the 3 wards (Figure 2). At baseline, 2 of the 11 HCFs had scores of less than 20% and 3 had between 35% and 41%. By end line, 7 facilities met all the assessment water criteria and the rest had total scores between 96% and 98% (Figure 4a).

Water improvements varied by facility and included increased water storage capacity, increased availability of water within the facilities, and increased number of water points in priority areas. MCSP provided all the facilities with a ceramic water filter station or bottled water dispenser in the 3 evaluated wards.

### Sanitation

The sanitation standard contributes 6 of the 100 points of the Clean Clinic certification over 5 criteria. At end line, 4 HCFs met all 5 sanitation criteria, and 4 HCFs had a level of compliance between 89% and 95% (Figure 4b). CAPs had the most delays in compliance. One CAP received a final score of 37% because its emergency room restrooms were not separated or signaled by gender, lacked accessibility for those with mobility issues, and were not clean.

Improvements to sanitation quality included rehabilitation of broken or shuttered sanitation facilities and adding in limited mobility access. All of the HCFs improved their restroom signage, cleanliness, privacy, and gender separation, as well as the placement of red bags for biological waste in each restroom.

**Figure:**
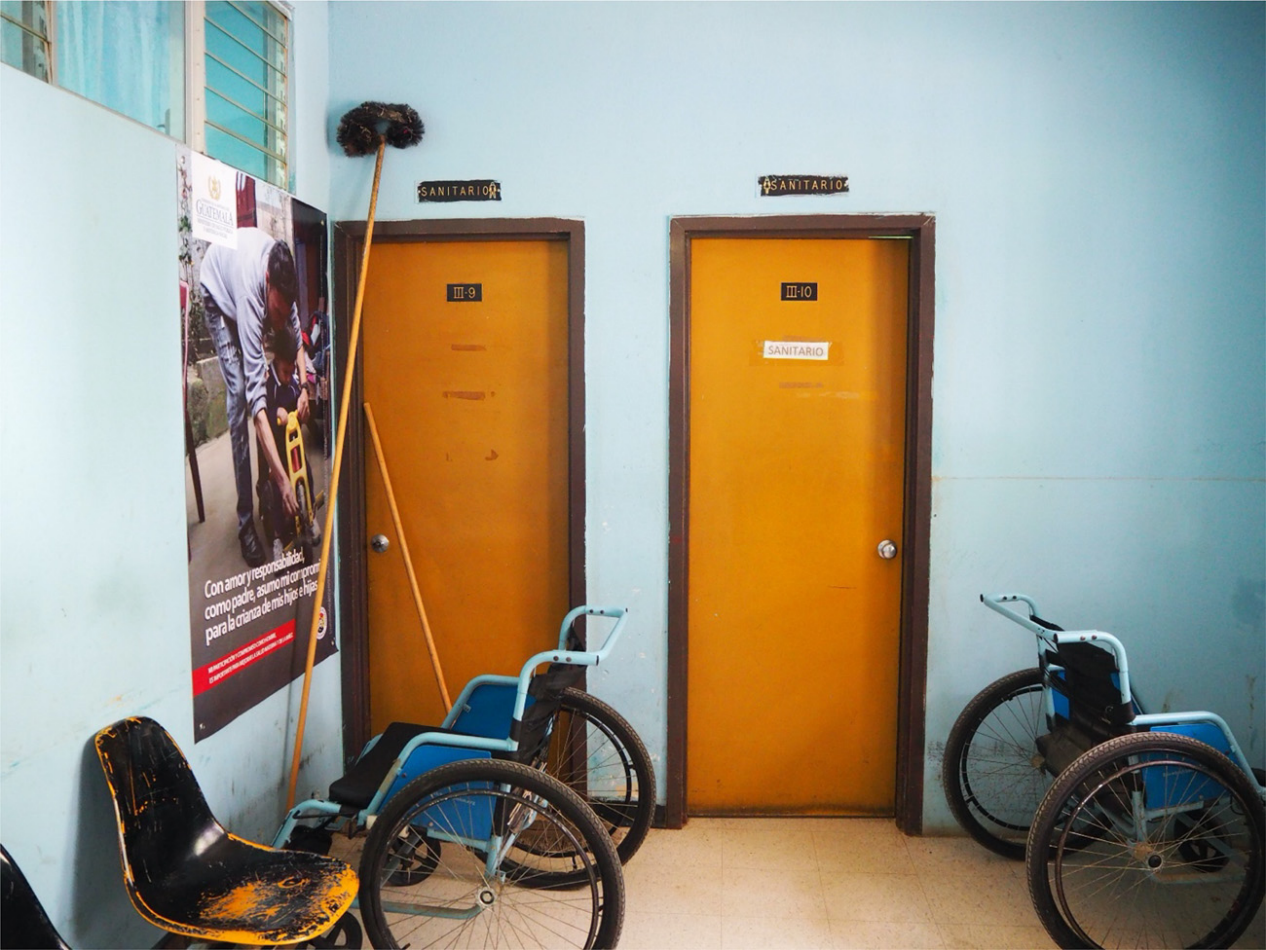
During the baseline assessment of health care facilities, it was common to find shuttered but functional latrines (left door), resulting in a reduced number of sanitation facilities and gender-segregated bathrooms.Photo Credit: © 2018 Jason Lopez/MCSP

### Hygiene

The hygiene standard has 13 criteria and contributes 28 of the 100 points of the Clean Clinic certification. At baseline, 7 of the 11 establishments had critically low compliance levels (below 34%). At end line, 1 hospital and 1 CAIMI complied with all criteria, and 1 CAP and 1 CAIMI scored above 95%. Six facilities reached compliance levels between 79% and 83% (Figure 4c).

The remaining hygiene gaps included the lack of showers with running water and lack of disposable towels for drying in delivery rooms and maternal recovery wards. In the delivery rooms, showers did not provide privacy or facilitate people with limited mobility and their size did not allow the option of having a companion if necessary.

In all of the facilities, handwashing stations were rehabilitated and availability of water, soap, and drying towels improved. Eight establishments closed gaps by fixing broken showers. In 1 CAP, conditions for handling and cleaning of bedding improved in the 3 wards. Additionally, 3 facilities improved their separation of the beds.

### Sterilization

The sterilization standard has 7 criteria and contributes 12 of the 100 points for Clean Clinic certification. HCFs had a compliance level above 60% in all services at first assessment. At end line, 9 facilities met all of the criteria and 2 achieved a score of 94% (Figure 4d).

Overall, HCFs improved the provision and use of sterile equipment (masks, scissors, clamps, and gowns).

### Waste Management

**FIGURE 5.**
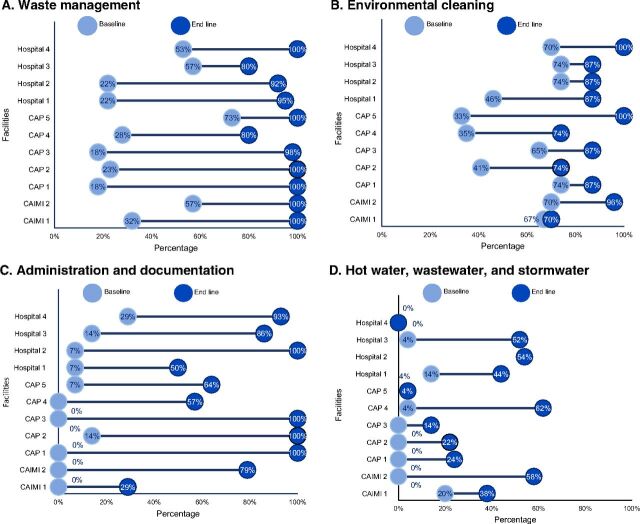
Baseline and End Line Assessment of Facility Compliance Level in Waste Management; Environmental Cleaning; Administration and Documentation; and Hot Water, Wastewater, and Stormwater Before and After Clean Clinic Approach Intervention, by Facility (N=11)

The waste management standard has 11 criteria and contributes 20 of the 100 certification points. At baseline, 7 facilities met less than 35% of the criteria; CAPs and hospitals had the lowest scores. At end line, 6 establishments met all waste criteria and the remainder reported levels of compliance above 80% (Figure 5a).

The activities for improving waste management included the correct separation of waste into red, black, and white bags and the addition of rigid containers for holding sharps in the assessment wards. MCSP also supported facilities with training cleaning staff on the correct use of personal protective equipment including lenses, masks, gloves, coveralls, and boots.

**Figure:**
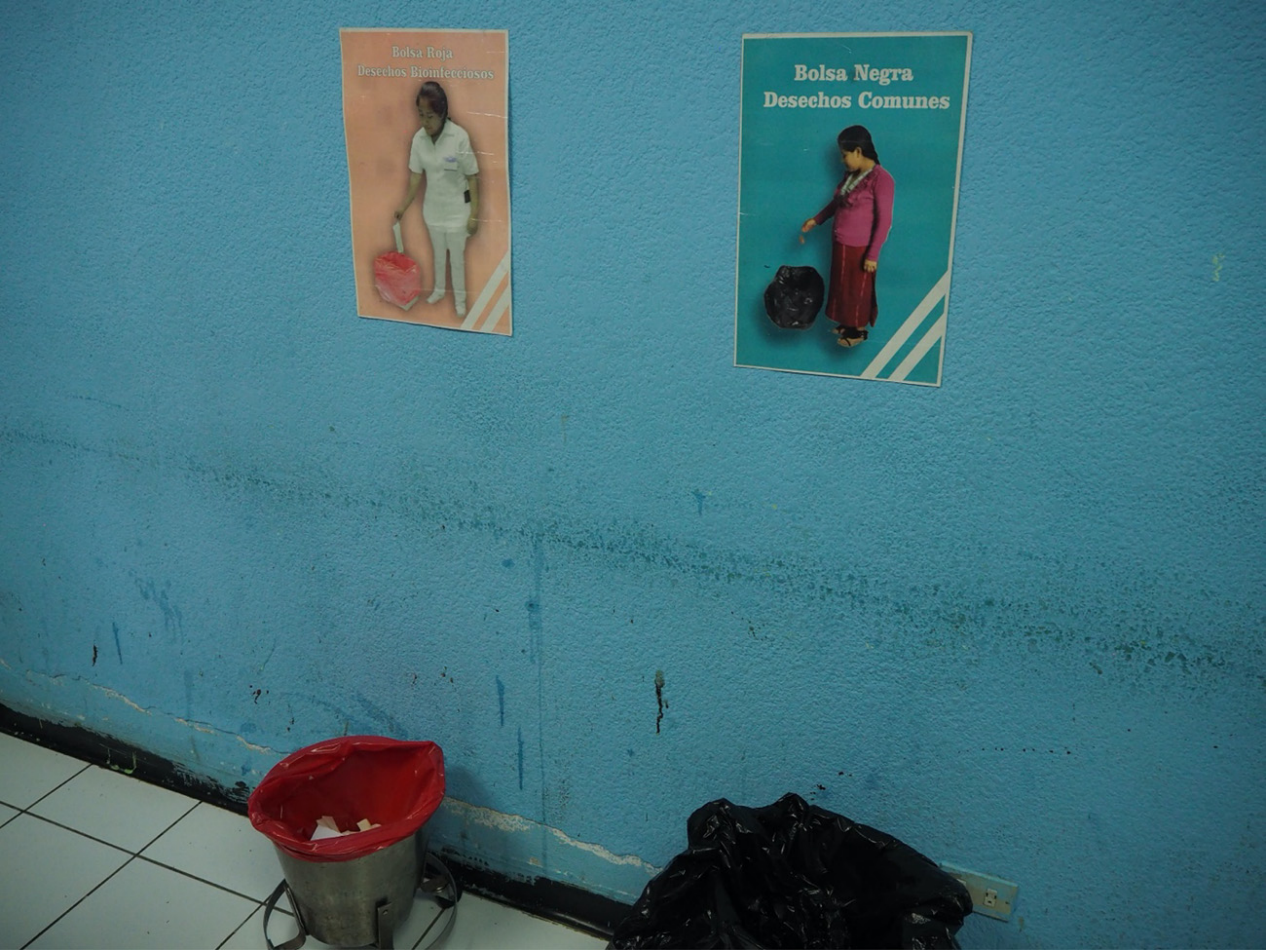
Nonverbal reliant signage and red biomedical waste bags were added to the maternal recovery ward in a hospital as a result of the Clean Clinic Approach intervention.Photo credit: © 2018 Jason Lopez/MCSP

Additionally, facilities identified temporary waste collection centers and began monitoring the correct separation of waste. In the 2 CAPs, nursing and custodial staff received direct training on the correct separation of waste according to the standards.

### Environmental Cleaning

The environmental cleaning standard consists of 9 criteria and provides 8 of the 100 certification points. At baseline, 7 HCFs met between 65% and 74% of the environmental cleaning criteria. Two hospitals had scores of 41% to 46%, and 2 CAPs had a compliance level of at or below 35% (Figure 5b). At end line, 2 facilities met all 9 cleaning criteria and 6 reached compliance levels between 87% and 96%.

Seven facilities developed and published cleaning control schedules and improved their compliance for scheduled reporting. Five facilities trained the custodial and nursing staff in the proper preparation and use of chlorine dilutions. Two CAPs and 1 CAIMI developed a manual on tasks and responsibilities for cleaning staff. Two CAPs worked with the district and municipalities to improve the availability of chlorine and detergent. One CAIMI improved storage, disposal of cleaning equipment such as mops, brooms, cleaners, detergent, and availability of chlorine.

### Administration and Documentation

The administration and documentation standards consist of 14 criteria and contributes 9 of the 100 points for certification. This standard had the lowest baseline scores with 10 of 11 facilities scoring 14% or lower. At end line, 4 facilities met all of the criteria and another 4 required improvement in their documentation processes (Figure 5c).

Activities for score improvement included placing posters and stickers with key messages on handwashing stations, waste disposal areas, and water sources and training custodial staff on cleaning and waste management procedures.

The facilities developed facility WASH improvement plans; a drinking water management protocol; a risk management plan for sanitation services; standard operating procedures for cleaning beds, cots, floors, sinks, and toilets; and a hospital solid waste management protocol. These plans and documents were shared among the participating HCFs during a knowledge-sharing workshop.

### Hot Water, Wastewater, and Stormwater

The hot water, wastewater, and stormwater standards consist of 11 criteria and provide 2 of the 100 points for certification. This standard presented the most challenges for closing gaps. At end line, only 1 CAP obtained scored 62%; 2 hospitals and 1 CAIMI scored higher than 50%. The remaining HCF’s compliance levels were less than 40%, and 1 hospital did not meet the criteria (Figure 5d).

Improvements included installation, rehabilitation, and maintenance of pipes and hot water in the showers. Personnel were also trained in the proper use of PPE for wastewater management, and compliance with tetanus vaccination schemes were verified and managed by the staff.

## DISCUSSION

The Guatemala CCA intervention demonstrated that the intervention could be implemented in a short period of time with limited resources to achieve quality improvements in WASH services. At the end of the intervention, all facilities had improved their levels of WASH services by both national and international standards.

Furthermore, the CCA provided valuable insights into the realities of WASH conditions and practices in HCFs in the Western Highlands of Guatemala and the risk that inadequate conditions pose to individual health and the provision of high-quality health care services.

The CCA provided valuable insights into realities of WASH and the risk that inadequate conditions pose to health.

Participating HCFs made substantial incremental improvements and achieved Clean Clinic certifications. WASH general management standards improved; toilets and sinks were in optimal condition with water, soap, and hand-drying towels; and common, special, and infectious waste was available and segregated where needed.

The categories with the most improvement, administration, and the least, cleaning, were most under the control of the HCFs. The reason for the lack of improvement in cleaning was mainly due to facilities being unable to develop cleaning schedules and protocols within the assessment period. However, the improvements in administration coupled with knowledge sharing among the facilities and incorporation into action plans could facilitate improving assessment scores.

According to feedback received during a knowledge-sharing workshop hosted by MCSP with participation from national and regional MSPAS and HCF staff, the contributing factors to the positive outcomes included integration of a steering team from the central level of the MSPAS; use of an easy to understand assessment tool for monitoring progress; in-service team trainings at the HCFs; technical support provided by MCSP WASH team; and involvement of local MSPAS authorities including hospital, district, and regional directors of health. Also, HCF staff stated that they were motivated to follow existing, but forgotten, IPC procedures and standards.

The 3 assessments were conducted at planned times. Based on the implementation team’s experience, assessments should be conducted at intervals of 2 months or more, allowing time for a thorough analysis of the findings and planning for continuous quality improvement based on the resources available and the time needed for execution. The improvements were subject to weekly facility-level monitoring and follow-up to verify progress and meet the monitoring and management needs with those responsible for each activity.

Clean Clinic teams were encouraged to seek solutions with the resources available at the facility as well as by reaching out to local stakeholders for support. Implementation and opportunity costs were maximized through community engagement and coordination with other local social actors such as the government, nongovernmental organizations, municipalities, and ancestral organizations.

### Sustainability

In May 2019, all 194 United Nations member states voted in favor of a World Health Assembly resolution for the improvement of water, sanitation, and hygiene (WASH) in health care facilities (HCFs). The resolution reflected the importance of improving and sustaining WASH services in improving quality of care, achieving universal WASH and health care coverage as part of the Sustainable Development Goals, and slowing the spread of antimicrobial resistance.^21^

To ensure the long-term sustainability of the CCA, we recommend considering several important factors. Engage communities in the Clean Clinic certification process to maintain existing improvements and mobilize resources for improvements that require them. Activation and operation of Clean Clinic teams in each facility should be formalized through administrative processes. Ensure integration of both WASH and IPC into any and all health care quality improvement efforts and improve WASH and IPC monitoring and data collection. Comprehensive data are needed for managers to make informed decisions on quality improvements and resource allocation. Data on health outcomes and associated costs will also allow managers to quantify any time and resources savings associated with improved WASH and IPC. Plan for operational resources, supplies, and infrastructure for WASH and IPC in the respective annual procurement plans of each facility along with their corresponding management, similar to how essential medicines are prioritized.

### Potential Use in Other Contexts

Ministry of Health authorities are interested in implementing, updating, using, and improving the assessment tool for measuring compliance with quality standards and are currently working toward national implementation of the CCA.

The CCA assessment tool and subsequent stakeholder feedback may also serve as the basis for developing Guatemala’s advanced service levels of the JMP standards to be defined by each country.

### Limitations

Due to the small sample size of the intervention (11 HCFs), the results are not considered generalizable. Although measurements were taken at the ward level, the HCF Clean Clinic certification was based on averages across the HCF. This may have had the unintended effect of masking changes within individual wards. The CCA assessment tool was the same regardless of the type of facility (hospital, CAP, or CAIMI). HCF staff noted that standards should be tailored to each facility level to accommodate their varying circumstances while still maintaining service-level standards.

The intervention focused on improving the availability of WASH services and supplies and did not collect data on intervention-related health outcomes. The intervention did not include direct patient and visitor engagement, which is a potential point of entry for hygiene improvements that may contribute to the continued demand for Clean Clinic certified facilities.

The institutional dynamics of the MSPAS constrained CCA implementation due to high staff turnover, slow administrative processes, lack of basic and minimum resources (soap and chlorine), and the limited human resources spread over many functions. The intervention did involve municipalities and local health committees that are responsible for facility infrastructure at the CAP and CAIMI level. No formal or public commitment was established with local municipal governments, resulting in their limited engagement in the process.

## CONCLUSIONS

The CCA process and tools facilitated a systematic way for HCFs to prioritize, make, and measure WASH quality of care improvements. Training facility staff was fundamental to improving quality standards, and involving medical and administration staff in joint analysis, coordination, and planning sessions was key to integration and teamwork. Further work is needed to increase involvement of local government and community members and to further adapt the process and tools. Additionally, the CCA tool can be revised to encompass primary care facilities and additional services within HCFs.

## Supplementary Material

19-00413-Lopez-Supplement_2.xlsx

19-00413-Lopez-Supplement_1.xlsx

## Notes

**En español**

**La implementación del Enfoque de Clínica Limpia mejora la calidad del agua, el saneamiento y la higiene en los establecimientos de salud en el Altiplano Occidental de Guatemala**

**Principales Conclusiones**

- Los servicios de agua, saneamiento, e higiene (WASH) y los suministros de prevención de infecciones son deficientes en los centros de salud guatemaltecos que proporcionan servicios de atención del parto.
- El Enfoque de Clínica Limpia resultó en mejoras significativas en muchos indicadores de preparación para WASH y la prevención de infecciones a pesar de muy poca inversión.
- El éxito y sustentabilidad del proceso del Enfoque de Clínica Limpia se basa en:
  - Establecer estándares claros, concisos y específicos para cada sala;
  - Orientar al personal a sus responsabilidades a medida que se aplican a los estándares provistos;
  - Recopilación de datos detallados a nivel de sala; y
  - Compartir los resultados de las mediciones con el personal de los establecimientos de salud, los gobiernos locales, y el público.

**Principales Implicaciones**

- El uso de intervenciones económicas como el Enfoque de Clínica Limpia puede ayudar a los establecimientos de salud a realizar y mantener mejoras efectivas e incrementales en la preparación para la prevención de infecciones.
- Para fomentar la futura expansión de WASH en los establecimientos de salud, los donantes, y los socios implementadores deben:
  - Colaborar con los ministerios nacionales de salud para revisar y actualizar las pautas nacionales para la prevención y el control de infecciones en los hospitales,
  - Promover la intervención entre los directores de hospitales y las direcciones regionales del ministerio de salud,
  - Asóciese con la administración de los establecimientos de salud para actualizar las herramientas de evaluación de acuerdo con el nivel de atención y el tipo de servicio/sala con consideración para el nivel básico de servicio, y
  - Amplíe la intervención para incluir más centros de atención médica y comparta los resultados públicamente.

**RESUMEN**

- **Contexto:** Los servicios de agua, saneamiento e higiene (WASH) son elementos esenciales para proporcionar servicios de atención médica seguros y mejorar la satisfacción del paciente y la búsqueda de atención de la salud. El Enfoque de Clínica Limpia (CCA) utiliza un proceso de 10 pasos para apoyar a los establecimientos de salud (ES) en la realización de mejoras incrementales y efectivas de limpieza y prevención y control de infecciones, sin depender de inversiones externas. Pusimos a prueba el CCA en Guatemala y evaluamos el grado en que contribuyó a las mejoras de calidad en WASH para prevención y control de infecciones.
- **Métodos:** Después de desarrollar una herramienta de evaluación adaptada al contexto guatemalteco, evaluamos 11 ES en 8 áreas técnicas y las calificamos en 79 criterios con un total de 100 puntos. Realizamos una medición de referencia (septiembre a octubre de 2018), una segunda medición (enero de 2019) y una medición final (febrero a marzo de 2019).
- **Resultados:** Los 11 ES mejoraron sus puntajes promedio en la sala de emergencias/general de 41 puntos al inicio del estudio a 87 puntos en la medición final, en base a una escala de 100 puntos. Para salas de parto, los puntajes aumentaron de 50 a 91 puntos y para salas de recién nacidos de 46 a 90 puntos.
- **Conclusiones:** El proceso y las herramientas del CCA facilitaron una forma sistemática para que los ES identifiquen, prioricen, realicen y midan las mejoras en la calidad de la atención de WASH. La capacitación del personal de las instalaciones fue fundamental para mejorar los estándares de calidad, y la participación del personal médico y administrativo en sesiones conjuntas de análisis, coordinación y planificación fue clave para la integración y el trabajo en equipo. Se necesita más trabajo para aumentar la participación de los gobiernos locales y los miembros de la comunidad y para adaptar aún más el proceso y las herramientas.

**Peer Reviewed**

**First published online:** May 21, 2020
